# Usability and Implementation Considerations of Fitbit and App Intervention for Diverse Cancer Survivors: Mixed Methods Study

**DOI:** 10.2196/60034

**Published:** 2025-02-24

**Authors:** Zakery Dabbagh, Reem Najjar, Ariana Kamberi, Ben S Gerber, Aditi Singh, Apurv Soni, Sarah L Cutrona, David D McManus, Jamie M Faro

**Affiliations:** 1 Department of Population and Quantitative Health Sciences, University of Massachusetts Chan Medical School, 55 N Lake Ave, Worcester, MA, 01655, United States, 1 (774) 455-3744; 2 Department of Medicine, University of Massachusetts Chan Medical School, Worcester, MA, United States; 3 Center for Healthcare Organization and Implementation Research, Bedford VA Healthcare System, Bedford, MA, United States

**Keywords:** physical activity, cancer survivor, wearable device, smartphone app, diverse, Fitbit, wearable, feasibility, usability, digital health, digital health method, breast cancer, Hispanic, women, mobile health, activity tracker, mHealth

## Abstract

**Background:**

Despite the known benefits of physical activity, cancer survivors remain insufficiently active. Prior trials have adopted digital health methods, although several have been pedometer-based and enrolled mainly female, non-Hispanic White, and more highly educated survivors of breast cancer.

**Objective:**

The objective of this study was to test a previously developed mobile health system consisting of a Fitbit activity tracker and the MyDataHelps smartphone app for feasibility in a diverse group of cancer survivors, with the goal of refining the program and setting the stage for a larger future trial.

**Methods:**

Participants were identified from one academic medical center’s electronic health records, referred by a clinician, or self-referred to participate in the study. Participants were screened for eligibility, enrolled, provided a Fitbit activity tracker, and instructed to download the Fitbit: Health & Wellness and MyDataHelps apps. They completed usability surveys at 1 and 3 months. Interviews were conducted at the end of the 3-month intervention with participants and cancer care clinicians to assess the acceptability of the intervention and the implementation of the intervention into clinical practice, respectively. Descriptive statistics were calculated for demographics, usability surveys, and Fitbit adherence and step counts. Rapid qualitative analysis was used to identify key findings from interview transcriptions.

**Results:**

Of the 100 patients screened for eligibility, 31 were enrolled in the trial (mean age 64.8, SD 11.1 years; female patients=17/31, 55%; Hispanic or Latino=7/31, 23%; non-White=11/31, 35%; less than a bachelor’s degree=14/31, 45%; and household income <US $75,000=11/31, 35%). The mean (SD) years since diagnosis was 7.1 (8.2), and the two most frequent cancer diagnoses were prostate (9/31, 29%) and breast (4/31, 13%) cancer. Participants provided positive feedback on the MyDataHelps app usability; the overall app quality received a mean score of 3.79 (SD 0.82) on a 5-point Likert scale (1=worst, 5=best). Interviews with 10 patients yielded four themes: (1) Fitbit and app setup was easy but the research team provided assistance, when needed, which was helpful, (2) motivational messages within the app were not memorable, (3) step counts and Fitbit notifications were motivating, and (4) medical professionals viewing their data were acceptable. Interviews with 5 cancer care clinicians yielded four themes: (1) some patients used wearables but rarely discussed data with clinicians; (2) activity trackers can be helpful to motivate patients and keep them accountable; (3) objective activity measures—similar to BMI, weight, and blood pressure— that they can track over time and refer to afterward were preferred; and (4) training and systematic processes to view these data as part of active workflow were desired.

**Conclusions:**

Implementing a remotely delivered, light-intensity physical activity program was feasible and acceptable in a sample of diverse cancer survivors. Future studies should consider registry-based methods and work with clinicians to engage hard-to-reach survivor populations who have low physical activity levels and disproportionately high adverse health outcomes.

## Introduction

The health benefits of physical activity for cancer survivors are widely known. Yet, few survivors are active during (<10%) and after cancer treatment (20%‐30%) [1,2]. Per the American Heart Association recommendation, cancer care clinicians should provide phsyical activity referrals to prevent cardiovascular disease in survivors [3]; however, clinicians do not always suggest these referrals [4]. Notably, acknowledging an individual’s unique perspective and offering choices rather than referrals to one singular program have been shown to increase effectiveness [5,6].

There has been a recent increase in the number of physical activity programs that exist for survivors. Virtual group programs have appeared as an option that is preferred by some survivors, allowing them the social support they seek in a group format with the convenience of participating in a program from one’s own home [7,8]. In a previous study, clinic-based referrals to group in-person and group virtual programs were acceptable to cancer survivors, but some also expressed a desire for a nongroup digital program option [8]. In cancer survivors, digital health programs can be effective in promoting physical activity and reducing participation barriers [9]. However, engaging diverse groups of survivors in digital programs continues to be a challenge [10], along with sustainably integrating digital programs into clinical survivorship care [11]. Ninety percent of US adults own a smartphone and 40%‐60% a wearable device [12,13]. As digital health programs are becoming increasingly more accessible, more strategies are needed to increase the reach of these programs, particularly as the number of cancer survivors grows annually [14].

This pilot study differs from past research in several ways. First, most prior digital health trials used pedometer-based interventions as opposed to Fitbit-based interventions [15], and many of these studies incorporated counseling and other forms of support in their interventions [15]. Additionally, the majority of trials using wearable physical activity trackers with cancer survivors have been done in homogenous populations, such as White, college-educated [10], and survivors of breast cancer [10,15]. Past evidence on the optimal frequency and timing of SMS text messages in this context is lacking and what evidence there is has been inconclusive [10]. Finally, very few studies have focused on older adults, who comprise the majority of cancer survivors in the United States [10].

Thus, the purpose of this study was to test a previously developed mobile health system in a diverse group of cancer survivors with the goal of refining the program and setting the stage for a larger future trial [16,17]. We tested the feasibility of using a wrist-based wearable activity tracker (Fitbit) and smartphone app dyad with survivors, including seeking feedback on survey data collection and message prompts within the app. We also sought feedback from our clinical partners to better understand the implementation of referrals to a digital health program and perspectives on accessing patient-generated health data from these programs.

## Methods

### Ethical Considerations

This study took place at the University of Massachusetts Chan Medical School with remote recruitment, allowing participants to partake in study activities outside of the institution. Procedures were approved by the University of Massachusetts Chan Medical School Institutional Review Board (#H00023545) and informed consent was obtained from each participant. All participant data were deidentified and study data were kept anonymous. Participants received US $25 compensation for participation in the pilot and US $25 for participation in a follow-up interview. The study was registered at ClinicalTrials.gov (NCT05417438).

### Study Design

This study used a longitudinal, nonrandomized, multilevel mixed methods approach, involving both quantitative and qualitative data collection (explanatory sequential design) from enrolled patients and cancer care clinicians between January 2023 and July 2023. Cancer survivors were enrolled in a 3-month Fitbit or smartphone app intervention. The convenience sample size was not calculated, as this was a feasibility pilot study. Outcome data for the intervention included Fitbit adherence and step counts over 3 months, usability ratings of the MyDataHelps app at 1 month, and follow-up interviews. No data were collected regarding the usability of the Fitbit: Health & Wellness app, as the app was primarily used to continuously synchronize Fitbit data with the MyDataHelps app. Clinician semistructured interviews were conducted following the data collection period for the intervention (June-July 2023).

### Recruitment

Eligibility criteria for the pilot study included having a past cancer diagnosis, owning a smartphone with internet access, and being deemed appropriate to participate by a medical professional as necessary. Recruitment was first conducted using paper flyers posted throughout clinics in the UMass Memorial Health network. The flyer provided interested participants with the study team’s email address and telephone number to call and SMS text message to discuss the study further. Potential participants were additionally identified by extracting data directly from the electronic health record (EHR) data hosted in the UMass Chan Data Lake. Next, potential participants were sent a letter containing information about the study and instructions to call to opt out of further contact. Two weeks after being mailed, potential participants who did not opt out were called and asked if they were interested in joining the study. Potential participants who expressed interest were screened over the phone. Approval to participate in the study was sought from clinicians for potential participants based on responses in the Physical Activity Readiness Questionnaire (PAR-Q), a 7-step questionnaire screening for evidence of risk factors during moderate physical activity and reviewing for family history and disease severity [18]. Potential participants were then sent a secure email through REDCap containing a link directing them to an electronic consent form. After going through the electronic consent form with the study team over the phone, eligible potential participants who were interested consented using their electronic signature.

### Statistical Analysis

#### Healthy History, Demographics, and Physical Activity Readiness

Participants self-reported their age, sex, education level, race, ethnicity, date of last cancer diagnosis, and type of cancer via a telephone screening process with the study team. Physical activity readiness was assessed using the PAR-Q. Any “yes” response prompted approval to be sought from the participant’s primary care provider or cancer care clinician. Once enrolled, patients completed additional health history baseline questionnaires through surveys delivered in the MyDataHelps app.

#### Fitbit Adherence and Step Count

Physical activity data were adjusted for a number of valid wear days. Consistent with prior studies [19-21], valid wear days were defined as those with a daily step count of 1500 or greater as measured by the Fitbit activity tracker.

#### App Usability

The Mobile App Rating Scale (MARS) was used to assess the acceptability of the MyDataHelps smartphone app at 1 month through a survey delivered in the MyDataHelps app. Specific assessments of app functionality and aesthetics included ease of use, navigation, visual appeal, performance, graphics, and layout, as well as overall app quality [22]. Items were rated by participants using a 5-point Likert scale from 1=inadequate to 5=excellent. Sample questions included “how easy is it to learn how to use the app?” and “how clear are the menu labels or icons and instructions?” The MARS was scored using a mean for each category. The MARS has demonstrated internal consistency (α=.9) and interrater reliability (intraclass coefficient=0.79) [22,23].

#### Participant and Clinician Acceptability

Enrolled participants completed semistructured interviews (Multimedia Appendix 1) after the 12-week intervention to assess their experiences during the study, including areas of improvement. Health care clinicians’ perceptions on integrating this program into clinical practice were also assessed through semistructured interviews.

### Protocol for Intervention

The intervention protocol, including the MyDataHelps app, was previously user-tested in a healthy cohort [24]. All participants received a Fitbit Charge device (versions 2 and 5) to keep as part of their compensation for participating. Following consent, participants were mailed their Fitbit along with paper instructions for device and app setup (including a unique study identifier) and were provided technical support over the phone by the study team. Study participants downloaded 2 apps onto their phones: Fitbit: Health & Fitness and MyDataHelps. Upon enrollment, participants were instructed that the standard goal steps/day set by Fitbit is 10,000 steps. They were told they could modify their step-count goal based on what their current activity level was and what they felt was achievable. Participants were instructed to wear their Fitbit for a total of 3 months, during which time they received standard Fitbit: Health & Fitness push notifications to their Fitbit devices, such as alerting them of achievement of goal steps per day, time spent in active heart rate zone minutes (if participants enter a high-intensity workout zone), and movement reminders (to get up and move if inactive for a period). These messages are preset by Fitbit to be delivered to participants based on their individual data.

**Figure 1.**
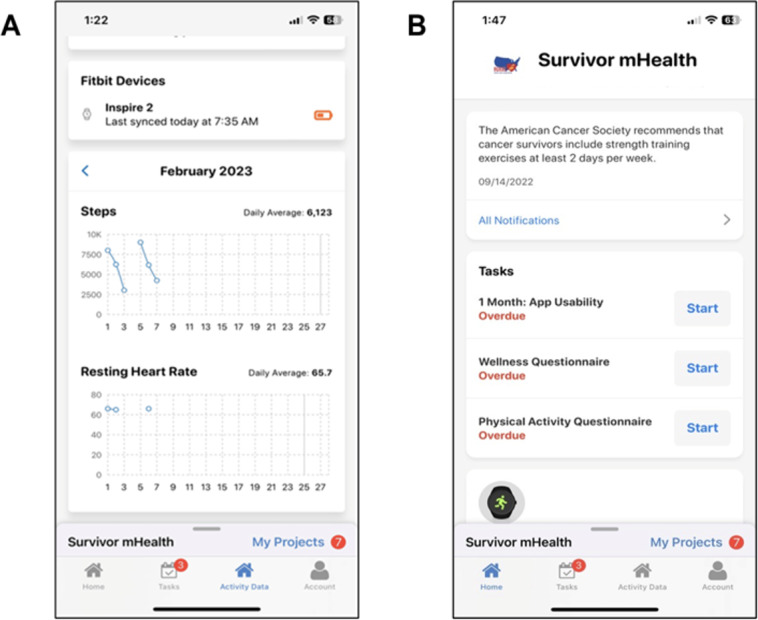
MyDataHelps app displaying Fitbit data (A) and message notifications and survey tasks (B).

Participants’ smartphones also received push notifications from MyDataHelps to promote adherence to the study surveys, along with weekly push notifications of motivational messages (Figure 1). These messages were templated messages to inform participants about the benefits of exercise and strategies to incorporate exercise into their day, derived from the American Cancer Society (eg, “Research has shown that exercise is not only safe and possible during cancer treatment, but it can improve how well you function physically and your quality of life” and “End your exercise session with stretching or flexibility exercises. Hold a stretch for about 15 to 30 seconds and relax. Examples of stretching are reaching overhead, deep breathing, and bending over to touch your toes so that you relax all the muscle groups”) [25].

Participants who experienced technical challenges or difficulties adhering to study protocols (eg, syncing Fitbit, completing surveys) were contacted by phone to troubleshoot issues. Through the MyDataHelps app, participants completed surveys 24 hours, 1 month, and 3 months after enrollment. Participants also received push notifications alerting them that the surveys were available within the app (Figure 1). Participants’ Fitbit devices were synced to the MyDataHelps app. Once participants registered for MyDataHelps and synced their Fitbit: Health & Wellness account, data collection from the Fitbit activity tracker began. The study team viewed activity and survey completion data weekly and conducted outreach to participants with missing data. We continued to reach out to participants, including giving them an opportunity to complete their 3-month survey. Those who did not complete their 3-month survey were considered lost to follow-up. After participants completed all the tasks required for the study, they were allowed to keep their Fitbit and were provided a US $25 Amazon gift card.

### Protocol for Qualitative Feedback From Participants and Cancer Care Clinicians

Upon completion of the study tasks, participants were offered the option to take part in a 30-minute semistructured Zoom interview. Participants who took part were compensated with a US $25 Amazon gift card (in addition to the gift card provided for the completion of study tasks). Interview guides were developed by the study team using an iterative process of pretesting. The interview guide consisted of questions asking their perceptions of the program overall, as well as asking them ways to improve the current list of messages they received within the app (Multimedia Appendix 2). These guides were revisited after interviews to determine if any additional questions needed to be added, removed, or probed further. Cancer care clinicians, specifically advanced practice providers, were recruited via pre-existing relationships with the study team and through the survivorship coordinator. The survivorship coordinator emailed cancer care clinicians involved in survivorship care information about the study and about participating in interviews. Participating clinicians were asked to complete a 30-minute Zoom interview using a structured interview guide. The guide was designed to discuss implementing physical activity referrals to digital health programs into clinical workflow, along with preferences for reviewing those data. Again, the interview guide was revisited after each interview for revisions. The survivorship coordinator also provided feedback on the interview guides throughout the process. Zoom interviews were conducted by a trained member of the research team in conjunction with the study principal investigator and recorded and transcribed electronically using an institutional review board–approved software.

### Data Analysis

Quantitative analyses for demographics, usability surveys, and Fitbit data were completed using R 4.3.2 (The R Foundation) and Microsoft Excel. Analysis of Fitbit step count data (mean and SD of steps per day each week) was done on valid days, in which valid days were defined as those with a step count of 1500 or greater [20-22]. As such, the first valid day of Fitbit use was the first day the participant walked 1500 or more steps while wearing Fitbit, and data collection from Fitbit concluded 90 days following the first valid day. Any days with fewer than 1500 steps within the 90-day time frame of study participation were considered missing. Qualitative data were analyzed using rapid qualitative analysis [26]. This was done by first creating a matrix in Excel after the participants’ interviews were completed. The matrix had a row for each participant and columns for each domain that corresponded with the interview guide. Domains included “experiences with patients and wearables devices,” “when to integrate program into clinical practice,” “preferences to clinical team viewing data,” and “support needed for clinicians (eg, EHR staff) or patients.” The study team (JMF, RN, AK, and ZD) then met to code the interview transcripts for 2 participants in each group, and then individually coded the remainder on their own. The study team met regularly to conduct data and coding checks and resolve any discrepancies in coding or domain assignment. The team noted the common themes between the participants’ responses to each domain and identified key findings of the qualitative interviews. Each key finding discusses the main common themes within the qualitative interviews that align with the domains.

## Results

### Overall Results

**Figure 2.**
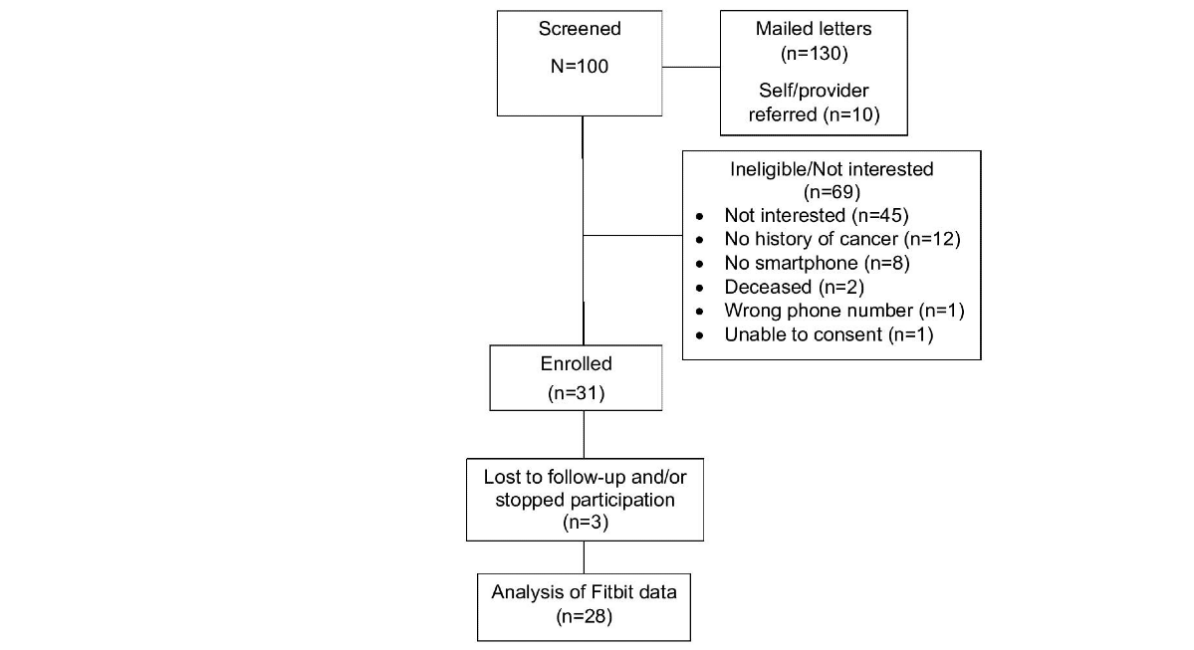
CONSORT (Consolidated Standards of Reporting Trials) flow diagram.

Figure 2 presents the flow of participants from contact to the conclusion of the 12-week intervention. The study team mailed letters to 130 potential participants and had 10 potential participants either self-refer or were referred by their clinician. Of the 100 potential participants who were screened, 31 participants enrolled in the trial, achieving a response rate of 31%.

Among the 31 participants enrolled in the intervention, the mean age was 64.8 (SD 11.1) years, 17 (55%) identified as female, 14 (45%) had less than a bachelor’s degree level of education, 7 (23%) identified as Hispanic or Latino, and 11 (35%) identified as non-White (Table 1). The top two cancer diagnoses were prostate (9/31, 29%) and breast (4/31, 13%), and the mean years since diagnosis was 7.1 (SD 8.2).

**Table 1.** Enrolled patient baseline demographics (N=31).

Variables		Values
Age (years), mean (SD)		64.7 (11.1)
**Sex, n (%)**		
	Male	14 (45)
	Female	17 (55)
**Education level, n (%)**		
	High school or less	7 (23)
	Some college	7 (23)
	Bachelor’s degree	6 (19)
	Advanced college degree	11(35)
**Race, n (%)**		
	White	20 (65)
	Black or African American	10 (32)
	Other	1 (3)
**Ethnicity, n (%)**		
	Hispanic or Latino	7 (23)
	Not Hispanic or Latino	24 (77)
**Household income in US$, n (%)** ^**a**^		
	>75,000	18 (62)
	50,001-75,000	4 (14)
	25,000-50,000	5 (17)
	<25,000	2 (7)
**Cancer type, n (%)^b^**		
	Gynecologic	2 (6)
	Thyroid	2 (6)
	Skin	2 (6)
	Colon	1 (3)
	Prostate	9 (29)
	Breast	4 (13)
	Throat	2 (6)
	Kidney	1 (3)
	Blood	2 (6)
	Don’t know	2 (6)
Years since diagnosis, mean (SD)		7.1 (8.2)

a Due to missing responses, n=29.

b Due to missing responses, n=27.

### Fitbit Adherence and Activity

Physical activity adherence during the 12-week intervention was assessed using Fitbit data (Table 2). Based on this measure, the mean daily step count was 7219 (SD 4418) at baseline and 6687 (SD 3183) at 12 weeks.

**Table 2.** Mean (SD) number of valid days of adherence to wearing the Fitbit activity tracker and mean (SD) steps/day during the 12-week intervention period among cancer survivors (n=28).

Intervention week	Number of valid days/week, mean (SD)	Steps/day, mean (SD)
1	1.93 (2.61)	7218.61 (4417.71)
2	4.78 (2.83)	6447.97 (3405.71)
3	5.30 (2.52)	6200.9 (3382.68)
4	5.22 (2.61)	6645.24 (3607.05)
5	5.04 (2.89)	6782.65 (3901.27)
6	4.85 (2.74)	6639.79 (4072.6)
7	5.11 (2.53)	6570.96 (3950.37)
8	5.07 (2.89)	6628.75 (3745.22)
9	4.70 (3.01)	6871.6 (4134.65)
10	4.69 (3.17)	6598.36 (3481.8)
11	4.77 (2.96)	7384.56 (4157.78)
12	4.08 (2.62)	6686.95 (3183.27)

### Mobile App Rating Scale

At 1 month, 25 participants completed the MARS usability scale responding to questions about the MyDataHelps app functionality, appearance, and overall rating on a scale of 1 to 5 (1=worst and 5=best; Table 3). The highest-rated category was “performance” with a mean score of 4.36 (SD 1.02), and the lowest-rated category was “ease of use” with a mean score of 3.79 (SD 1.22). The overall app quality received a mean score of 3.79 (SD 0.82).

**Table 3.** MARS (Mobile App Rating Scale) usability scores of the MyDataHelps smartphone app; ratings range from 1=worst to 5=best.

Variable and question	Values, mean (SD)
Performance: How accurately/fast do the app features (functions) and components (buttons/menus) work?	4.36 (1.02)
Ease of use: How easy is it to learn how to use the app? How clear are the menu labels/icons and instructions?	3.79 (1.22)
Navigation: Is moving between screens logical/accurate/appropriate/uninterrupted? Are all necessary screen links present?	4.21 (1.22)
Interactions: Are interactions (taps/swipes/pinches/scrolls) consistent and intuitive across all components/screens?	4.13 (1.05)
Layout: Is the arrangement and size of buttons/icons/menus/content on the screen appropriate or zoomable if needed?	4.04 (1.02)
Graphics: How high is the quality/resolution of graphics used for buttons/icons/menus/content?	3.80 (0.85)
Visual appeal: How good does the app look?	3.84 (0.78)
Overall rating: What is your overall star rating of the app?	3.79 (0.82)

### Qualitative Feedback

Ten participants completed 3-month follow-up interviews assessing their overall experiences in the program. Four themes were identified from our analyses, and illustrative quotes can be seen in Table 4. Overall, participants felt the Fitbit activity tracker and the MyDataHelps app were easy to use but also benefited from the assistance of the research team. The weekly motivational messages within MyDataHelps were not very memorable and were often confused with the daily push notifications sent directly to the Fitbit activity tracker. For example, when asked about the content of the MyDataHelps weekly messages, a participant responded, “I didn’t pay a whole lot of attention to them. I know I got ‘em maybe daily or every other day.” However, participants noted overall that the messages and notifications motivated them to be more active and accrue additional steps. Lastly, most participants were comfortable with and liked the idea of sharing their physical activity data with their clinicians.

**Table 4.** Enrolled patient feedback from qualitative interviews.

Theme	Illustrative quotes
Fitbit and app setup were easy and assistance from the research team was helpful.	“I didn’t find it [Fitbit and MyDataHelps setup and navigation] to be a problem at all once you walked me through it.” “The information provided was pretty self-explanatory, even though I’m not very computer-savvy and never had a Fitbit.”
Motivational messages within the app were not memorable.	“They weren’t sent often. Not, not particularly unless I set it up wrong. I think if I had gotten something on a weekly or even a daily [basis], it would motivate me.” “Well, the messages, if any, were, I believe...I forget how I was able to access them...I’m trying to think of a specific message.”
The step counts and Fitbit notifications to be active were motivating.	“Tracking my steps was helpful, especially given how I’ve changed my lifestyle from working full-time to finally being home most of the time. And I had to make sure I put some time into taking steps or helping my health progress.” “Before, I never actually paid attention to like, ‘Okay, I’m gonna do this many steps today, or I’m gonna do this.’ And that was pretty cool that it gave that option to keep track of all that. And if I beat my goal from the day before, it would let me know. It would tell me, ‘Congratulations!’ which was kind of cool.”
Patients were comfortable with and liked the idea of medical professionals seeing their data.	“I did tell my oncologist that I was doing this study and I also spoke with my pulmonologist...And I told my primary care, and she said, “Well, that’s interesting.” I said, “You know the thing is, you never know what [information] is going to come along that might be helpful to you in the long run.” “I would like that because it’s able to connect some of the dots, right? They’re only seeing, you know, the medical side of it, but they can’t see your everyday activity.”

Five advanced practice providers specializing in oncology completed interviews to assess their perceptions of the digital health program and workflow integration. All 5 clinicians were female, had been practicing in their clinic for >1 year, and treated either disease-specific patients (breast, gastrointestinal, or genitourinary) or patients of all diagnoses. Clinicians were from one academic hospital (n=4) and one community hospital (n=1). Four themes were identified with illustrative quotes represented in Table 5. Clinicians noted some patients use wearables but rarely bring them up during their clinic visits. They also expressed that goal setting may be helpful for their patients and thought it may be helpful to have objective metrics of physical activity to track over time in lieu of standard weight and BMI clinical measures. Most clinicians wanted more education and training on physical activity and wearable device programs and better standard operating procedures integrated into their workflows.

**Table 5.** Clinician feedback from qualitative interviews.

Theme	Illustrative quotes
Some patients use wearables but rarely discuss data with clinicians.	“I don’t know that it comes up all that often. Things have changed since Covid. I used to have tons of patients who would do this kind of thing like the Silver Sneakers-type program...Not too much beyond the generic, like, ‘I shoot for X number of steps a day and I usually [get] X.’”
Activity trackers can be helpful to motivate patients and keep them accountable.	“Some people are motivated by their internal motivations. And I think Fitbit still gives you that accountability and that trackability. I’ve had patients that have used pedometer-esque, Fitbit-like, applications, whether it’s their iPhone or something like that, and they’ll say, ‘Okay, I got this many steps.’ And I think that it does motivate them to be more active.”
Clinicians prefer objective measures similar to BMI, weight, and blood pressure that they can track over time and refer to afterward (eg, vital signs).	“Typically, what I use is their BMI...to say, ‘Oh, congratulations! Your BMI last visit was this and look what it is now. I can see that you have better energy, and your wellbeing seems to be improved as well.’ It could be helpful for that sort of reinforcing and motivating and monitoring portion.”
Clinicians want more training and systematic processes to view these data as part of active workflow.	“With training and guidance, if that was considered part of our scope, if we felt like patients were benefiting...yeah, absolutely...if it’s something that would help patients and is clinically appropriate depending on whatever training we got.”

## Discussion

### Principal Results

Our study found that deploying a Fitbit or app dyad remotely was feasible and acceptable in this convenience sample of diverse participants with histories of several types of cancers. Participant engagement, as indicated by mean valid wear days and mean daily steps each week, was highly consistent over the course of the 12-week intervention. This finding was similar to those of past trials in cancer survivors [27-29]. The main exception to this was that on average, participants had fewer valid wear days in the first week of the intervention, which, similar to other trials, can be attributed to an initial adjustment period to wearing a Fitbit daily [30]. Though engagement was high, step counts remained relatively unchanged from baseline to 12 weeks. Lastly, several cancer care clinicians were interested in the ability to deploy such an intervention into their clinical practice for physical activity surveillance and interventions for their patients with cancer histories.

The MARS app ratings were similar to other physical activity monitoring app studies in that functionality components were rated higher than aesthetic components [24,31]. The high functionality ratings were supported in our qualitative feedback, with participants specifically noting the app being easy to use even if they themselves were not very tech-savvy. This is critical in the cancer survivor population, as 67% of US cancer survivors are over the age of 65, and this proportion is expected to grow to 74% by 2040. There are noted disparities in the use of digital health tools in advanced age [32]. However, participant qualitative feedback revealed the messages sent within the app were not very memorable. This may have been due to the message content, or possibly that they did not know where to view the messages on the MyDataHelps app dashboard. Several participants made more mention of the daily Fitbit device notifications being helpful. As motivational messaging has been shown to be effective in promoting physical activity [33,34] and was memorable to our participants on the Fitbit device, it is possible the notifications, along with vibrations, going directly to a patient’s device on their wrist are more noticeable than having to open an app to see the message. Despite notifications being reported as helpful, changes in step count over time were minimal. Though consistent with prior feasibility studies in this population [27,35], it will be critical to explore the effect of intervention components on step count over time in a large randomized trial. This includes exploring the addition of social support, a feature in Fitbit, and a feasible and acceptable intervention method for cancer survivors [35].

Qualitative feedback from participants yielded several important implications of recruiting, enrolling, and retaining patients throughout the study. The first important implication was that the MyDataHelps and Fitbit: Health and Wellness apps setup was straightforward and easy to follow. Some participants noted they were not tech-savvy, but they did not find it to be an issue. Participants also appreciated the help from the study team during the initial setup and to troubleshoot any issues that arose. To provide technical support, our study team used multiple methods to meet the needs of the participants. For most, this only entailed troubleshooting issues over the phone or SMS text message. Six participants additionally required videoconferencing and screen sharing to troubleshoot technical difficulties with a visual aid. Of the 6 participants, 4 chose to come in person for the study team to troubleshoot their issues. With additional assistance by video call or in person, these participants were also able to complete the study tasks quickly. Overall, the team found that participants greatly appreciated having options to connect and talk with them. The final theme from participants indicated they were comfortable sharing data with health care clinicians. Health care professionals can be critical in helping survivors engage in physical activity. Survivors reported a lack of physical activity guidance, prescriptions, and referrals from their care team as barriers to activity [36].

Clinicians revealed that patients rarely discuss wearable devices or data with them during their clinic visits. Oncology nurses have reported that patients lack interest in discussing physical activity with them. However, clinicians liked the notion of having long-term activity monitoring as a topic to discuss with patients in lieu of discussing weight or BMI. Physical activity independent of weight loss can improve health outcomes in individuals with obesity [37], and sedentary time, which is inversely correlated with physical activity levels, is an independent risk factor of cancer incidence [38]. Using a more neutral objective measure of physical activity rather than a more sensitive metric like weight or BMI may be more acceptable to discuss with patients who have weight concerns. To do this, clinicians expressed wanting more education and training on integrating physical activity digital health tools into their workflow as options for their patients. Lack of provider knowledge has been one of the most commonly reported barriers to the provision of physical activity promotion by cancer care clinicians to survivors [39]. Additionally, integrating wearable devices and subsequent data directly into clinicians’ workflow poses challenges. Prior studies have identified barriers to integrating devices into the EHR, maintaining privacy and confidentiality of patient data, lack of system interoperability and connectivity of wearable devices and health systems, and patient information or data overload [40].

### Limitations

This study has limitations to note. The small sample size is a statistical limitation. Similar to most studies done in the past, this study was limited to 3 months in duration; thus, there still remains little research regarding long-term outcomes from Fitbit-based interventions, including health outcomes [11,16]. As trials longer than 6 months have noted greater dropout rates over time [16], it will be important to explore this timeframe in future trials. Another limitation regarding the study design was the lack of a control group. Although this design has been used in similar feasibility trials, the lack of a control group limits the inferences we can make pertaining to the internal validity of the intervention. Though participant step counts were fairly consistent during the trial, this consistency could be attributed to having a highly motivated sample of individuals or to the Hawthorne effect and its impact on motivation and behavior. With regard to app usability, some participants reported confusing the SMS text messages delivered by the MyDataHelps app with push notifications sent by the Fitbit: Health & Wellness app, which may have affected their MARS usability score evaluation. Additionally, familiarity and comfort with navigating smartphone apps associated with wearable technology were not assessed. Notably, we relied on recruiting patients from an EHR patient registry list. This method may have allowed us to capture patients who would not otherwise have reached out to join the study, but also may have introduced selection bias and is less pragmatic than a program that is implemented within routine clinical workflow. While we examined step counts only, it should be noted that Fitbit tracks additional metrics of minutes and intensity of activity and time spent being sedentary that should be considered. Lastly, we ascertained that most of the clinicians wanted training, guidance, or knowledge of how to integrate these into their workflow. Given the busy workload of clinicians, it is possible that in practice they would prefer better reports and objective data to be provided, rather than actively taking part in new tasks added into their workflow. This should be examined in future trials.

### Conclusions

This study showed that a remotely delivered light-intensity physical activity program was feasible and acceptable in a sample of diverse cancer survivors. Future studies should consider registry-based methods and other strategies to engage hard-to-reach cancer survivor populations who are known to experience disproportionately high adverse health outcomes and low physical activity levels. These strategies should also be directed toward making improvements to recruit and engage larger numbers of participants from diverse sociodemographic backgrounds with consideration to technology access and use. On an individual scale, since some participants may have been more intrinsically motivated than others, future trials will benefit from assessing the underpinnings of participant motivation. Lastly, future trials should place emphasis on clinic implementation, including the quality of and method of delivery for reports, given the noted importance and use of the wearable device and app integration by cancer care clinicians for their survivors to be motivated to engage in and sustain physical activity.

## Supplementary material

10.2196/60034 Multimedia Appendix 1 Patient and provider interview guides.

10.2196/60034 Multimedia Appendix 2 MyDataHelps app push notifications.

## Abbreviations

EHR: electronic health record
MARS: Mobile App Rating Scale
PAR-Q: Physical Activity Readiness Questionnaire
