# Supplementary material for: Usability and Implementation Considerations of Fitbit and App Intervention for Diverse Cancer Survivors: Mixed Methods Study
Source: JMIR Cancer. 2025 Feb 24;11:e60034. doi: 10.2196/60034 (PMC11875131; doi:10.2196/60034)
Supplement: Multimedia Appendix 1 [file cancer-v11-e60034-s001.docx]

| **Patient Interview Guide**  First, I will ask some questions about the Fitbit/MyDataHelps app set-up and navigation:   1. Thinking back to the start, how was the process of receiving the Fitbit and setting up the accounts/apps for you?    1. What things made it easier? What things made it challenging? Any suggestions on how we could make it easier for you? 2. How was the experience of wearing the Fitbit daily?    1. What issues, if any, did you face with that?    2. What issues, if any, did you face with opening the app daily to “sync it”? 3. With the MyDataHelps app, how was your experience navigating through it?    1. How were your experiences with completing surveys? Was anything challenging? How could we make those better for you?   Now, I will ask some questions about the program itself:   1. What did you find most helpful from Fitbit/MyDataHelps app (i.e. steps, sleep, active minutes)? 2. What types of support, if any, would be helpful in setting up goals for yourself, or in understanding the data you saw?    1. Examples might include more guidance on goal setting, in paper format, or guidance from a healthcare provider or a health coach. 3. How was your experience with receiving messages about physical activity weekly?    1. What are your thoughts on how often they were sent? The content of the messages? What kinds of messages would you find to be helpful to you?       1. Examples might be prompting you to be active, congratulating you for being active, or about others like you and their success being active. 4. Lastly, what are your thoughts on having your clinical care team prescribe a Fitbit/app program?    1. How would you feel about your cancer care provider or PCP viewing your activity data?       1. What types of information related to this study would be helpful for them to know? For them to discuss with you?   *“Is there anything else you would like to add in that we haven’t discussed today?* |
| --- |
| **Provider Interview Guide**  First, I will ask some questions to ask your thoughts on integrating wearable devices, like Fitbits or Apple watches synced to apps, with your cancer patients.   1. What are experiences with your patients using wearable devices like Fitbits and app-based physical activity programs? 2. When and how do you feel it’s appropriate to offer it to patients? 3. How do you feel about being able to view participant data-generated from these programs?    1. Steps/day; Minutes of physical activity and sitting time?    2. How often would you want to see these data? 4. What would be the best way to integrate referrals to this type of program into your workflow? 5. What would be the best way to implement a program/dashboard for you to view patients’ data? Some examples might be in electronic medical record, Using prompts/alerts or in-basket notifications?   *“Is there anything else you would like to add in that we haven’t discussed today?”* |
