# Supplementary material for: Usability and Implementation Considerations of Fitbit and App Intervention for Diverse Cancer Survivors: Mixed Methods Study
Source: JMIR Cancer. 2025 Feb 24;11:e60034. doi: 10.2196/60034 (PMC11875131; doi:10.2196/60034)
Supplement: Multimedia Appendix 2 [file cancer-v11-e60034-s002.docx]

| **Weekly MyDataHelps App Push Notifications** | |
| --- | --- |
| **Notification Title** | **Notification Message** |
| **Randomly one of the following:**  The American Cancer Society recommends that cancer survivors:  - Take part in regular physical activity.  - Avoid inactivity and return to normal daily activities as soon as possible after diagnosis. – Aim to exercise at least 150 minutes per week.  - Include strength training exercises at least 2 days per week. | **Randomly one of the following**:  - Research has shown that exercise is not only safe and possible during cancer treatment, but it can improve how well you function physically and your quality of life.  - Too much rest can lead to loss of body function, muscle weakness, and reduced range of motion. So today, many cancer care teams are urging their patients to be as physically active as possible during and active after treatment. - Set short-term and long-term goals.  - Focus on having fun.  - Do something different to keep it fresh. Try yoga, dancing, or tai chi.  - Ask for support from others, or get friends, family, and co-workers to exercise with you.  - Use charts to record your exercise progress.  - Recognize and reward your achievements. - Cancer survivors may need to exercise less intensely and increase their workout at a slower rate than people who haven’t had cancer. Remember, the goal is to keep up as much activity as possible. Keep it safe, keep it fun, and make it work for you.  - If you were exercising regularly before you were diagnosed with cancer, you may need to reduce the intensity and length of your exercise sessions.  - Starting an exercise program can be a big task, even for a healthy person. It may be even harder for you if you have a chronic illness, especially if you weren’t used to exercising before your diagnosis. Start slowly and build up as you are able.  - The key is to keep your exercise program simple and fun. Exercise and relaxation techniques are great ways to relieve stress. Reducing stress is an important part of getting well and staying well.  - The more you exercise, the better you’ll be able to exercise and function. But even if planned exercise stops, it’s good to keep being active by doing your normal activities as much as you can. |

| **Survey Reminder Messages** | | | | |
| --- | --- | --- | --- | --- |
| **Notification** | **Condition** | **Time of Deployment** | **Notification Title** | **Notification Message** |
| New Surveys Available | Has new surveys | Day of deployment: morning or evening | New Survey(s) Available | New surveys are available to complete. Please open the MyDataHelps app to complete them! |
| Survey Reminder | Has incomplete survey(s) 1 week after deployment | 1 week after survey deployment: morning or evening | Complete Available Survey(s) | Please remember to complete your surveys! |
| Survey Thank You | Has completed all available survey(s) | Day after survey completion: morning or evening | Thank you for competing your surveys! | You have successfully completed your surveys. Thank you for being an essential part of this study! |
